# Identification of Secondary Nucleation Inhibitors of Amyloid‐β Aggregation by Cellular Selection of a SICLOPPS Library

**DOI:** 10.1002/cbic.202500908

**Published:** 2026-04-27

**Authors:** ByungUk Lee, Brian Flood, Emma Potter, Tina Wang

**Affiliations:** ^1^ Department of Chemistry University of Wisconsin‐Madison Madison Wisconsin USA

**Keywords:** amyloid, cyclic peptides, peptides, protein aggregation, split‐inteins

## Abstract

Alzheimer's disease is characterized by the accumulation of amyloid beta (Aβ) aggregates. Soluble oligomers Aβ oligomeric intermediates (AβOs) generated during aggregation are hypothesized to be a neurotoxic species. Many cyclic peptides have been developed to inhibit Aβ aggregation but primarily target Aβ monomers and fibrils; few cyclic peptides selectively recognize AβOs. We selected a library of >10^7^ cyclic peptides generated by the widely used split‐intein mediated circular ligation of peptides and proteins (SICLOPPS) strategy for binders of AβOs. These selections identified *cyclo*‐CRLISFF, which significantly delayed Aβ42 aggregation in vitro but displayed a mechanism inconsistent with inhibitors selectively targeting AβOs. To resolve this discrepancy, we tested whether intermediates formed during SICLOPPS cyclic peptide generation might also possess AβO binding activity. Our experiments showed that the CRLISFF sequence was active as an intein‐bound intermediate which selectively targeted AβOs by inhibiting the secondary nucleation step of the Aβ42 aggregation cascade. This intermediate has not been previously examined in studies employing SICLOPPS and may present a convoluting factor when using this technology to generate cyclic peptide libraries. The CRLISFF motif also retained activity when transplanted onto an unrelated protein scaffold, suggesting that SICLOPPS sequences may be compatible with peptide grafting strategies used to create protein‐based binders.

## Graphical abstract

Split intein mediated circular ligation of peptide and proteins mediated selection of 7‐mer cyclic peptide library for binding Amyloid‐β oligomers identified cyclic peptide sequences that greatly inhibited Aβ42 aggregation when cyclized in vitro and grafted as a loop sequence on proteinogenic scaffolds.

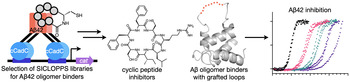

## 1 Introduction

Alzheimer's disease (AD) is an aging‐linked neurodegenerative disorder that affects millions worldwide with no cure to date [1]. AD pathology is characterized by the aggregation of amyloid beta (Aβ) into insoluble plaques in the brains of affected individuals [2, 3]. Decades of biophysical studies have elucidated the aggregation pathway of Aβ, where monomers self‐associate to form soluble oligomeric intermediates, which aggregate further to form mature amyloid fibrils [4]. Furthermore, fibril surfaces bind monomers and catalyze their conversion into oligomers and fibrils, a process known as secondary nucleation [5, 6, 7]. While the molecular mechanisms responsible for AD remains to be conclusively identified, soluble Aβ oligomeric intermediates (AβOs) have been hypothesized to be the major cause of neuronal toxicity [8, 9, 10]. Thus, agents that can target these species have garnered wide interest in the field [11].

Many antibodies and antibody mimetics that recognize AβOs have been identified, but large proteins suffer from limited bioavailability and may poorly penetrate the blood brain barrier [12]. An alternative strategy is to employ peptides, which are much smaller in size, to bind Aβ species [13]. In particular, macrocyclic peptides have drawn considerable interest due to their increased potency and stability relative to linear peptides [14, 15]. However, while many cyclic peptides that rescue Aβ aggregation through binding monomers or fibrils have been reported [16, 17, 18, 19, 20, 21, 22, 23, 24, 25, 26, 27, 28, 29, 30, 31], few AβO‐targeting species have been identified by comparison [34]. The dearth of structural information on AβOs present challenges to uncover binders to these species, especially through the rational design approaches that have been widely used to generate anti‐Aβ cyclic peptides.

An alternative to rational design is to select libraries of genetically encoded cyclic peptides for members with some desired activity. In vitro display methods such as mRNA and phage display have used this strategy to uncover peptide binders of immobilized amyloidogenic protein targets [36, 37, 38, 39]. However, AβOs are both unstable and heterogeneous, making it challenging to isolate them to use as epitopes for in vitro display. Cyclic peptide inhibitors can also be identified through cellular selections [40]. Here, biosynthesized cyclic peptide libraries are phenotypically assessed for their effect on cell growth or their ability to trigger genetically encoded circuits that sense some desired activity. An advantage of this strategy is that the target protein does not need to be isolated, making it suitable for identifying inhibitors of aggregation‐prone proteins. Cyclic peptides targeting α‐synuclein, mutant superoxide dismutase, hIAPP, and Aβ42 have been uncovered through cellular selections employing cyclic peptide libraries biosynthesized through the widely used split‐intein mediated circular ligation of peptides and proteins (SICLOPPS) strategy [16, 17, 41, 42]. However, these efforts have not yet been directed specifically toward oligomeric intermediates of Aβ.

Here, we investigated whether we could identify cyclic peptides that recognize AβOs using a strategy we recently developed to select for oligomer binders. We selected a library of > 10^7^ SICLOPPS cyclic peptides and identified a sequence, *cyclo*‐CRLISFF, that was active in cellular reporter assays. We initially observed strong dependence on splicing for *cyclo*‐CRLISFF activity using inactivating intein mutations previously employed to eliminate non‐splicing hits in SICLOPPS selections. Additionally, chemically resynthesized *cyclo*‐CRLISFF robustly suppressed Aβ42 aggregation when tested in vitro. However, *cyclo*‐CRLISFF inhibited the lag time of aggregation onset, behavior associated with monomer rather than oligomer binding. Our efforts to reconcile this discrepancy identified an intein‐bound SICLOPPS splicing intermediate of this cyclic peptide that was also active, both in our cellular assays and in in vitro Aβ42 aggregation assays, where it strongly suppressed secondary nucleation, consistent with an oligomer binding mechanism. This intermediate has not been examined in previous studies employing SICLOPPS, which have controlled for splicing dependency using mutations that pause splicing at other steps of the SICLOPPS cascade. We also discovered that CRLISFF is both active and retains behavior consistent with oligomer binding when grafted onto a completely different protein scaffold. Thus, cyclic peptide sequences uncovered using SICLOPPS may be active in a variety of contexts.

## 2 Results and Discussion

### 2.1 Selection for SICLOPPS Cyclic Peptides that Bind cCadC‐Aβ42 Oligomers

**FIGURE 1:**
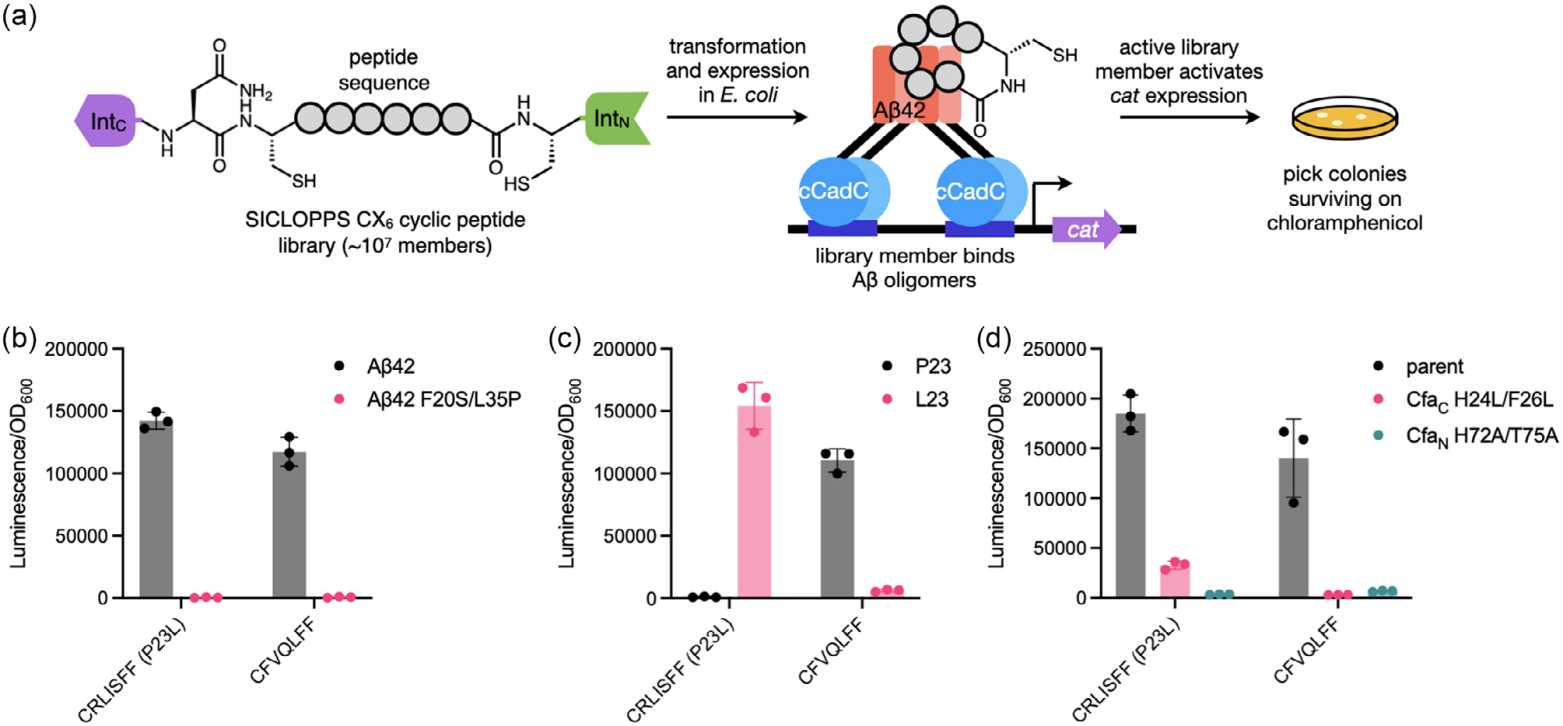
Selection for cyclic peptides that bind Aβ oligomers. (a) Overview of the cCadC‐Aβ42 growth‐based selection strategy for macrocyclic peptides that bind AβOs. (b) Effect of CRLISFF (P23L) or CFVQLFF co‐expression on *luxAB* transcriptional activation by cCadC fused to Aβ42 WT or the monomeric F20S/L35P mutant. (c) Effect of amino acid identity at position 23 on the activity of the CRLISFF or CFVQLFF sequences tested on cCadC‐Aβ42 WT. (d) Effect of intein splicing inactivating mutations on the activities of CRLISFF (P23L) and CFVQLFF tested on cCadC‐Aβ42 WT. (b–d) Data shows the mean and standard deviation of three biological replicates plotted individually.

We previously showed that an oligomerization‐dependent transcription factor, cCadC, can be used to detect binding to AβOs arising from the aggregation cascade in *E. coli* [43, 44]. In this strategy, proteins that bind to oligomeric cCadC‐Aβ42 fusions stabilize these otherwise transient species long enough to activate cCadC‐dependent gene transcription. Placement of a selection marker such as chloramphenicol acetyltransferase (*cat*) under the cCadC‐controlled *cadBA* promoter (P_*cadBA*_) thus enables the selection for AβO binders through resistance to the antibiotic chloramphenicol (Figure 1a).

**SCHEME 1:**
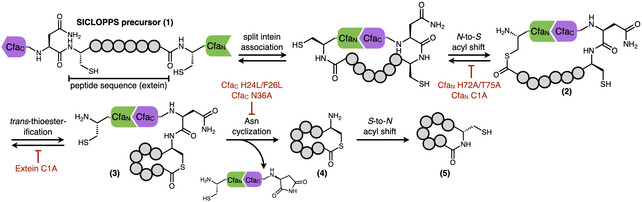
Mechanism of cyclic peptide formation by SICLOPPS. Mutations in the Cfa intein scaffold that pause SICLOPPS splicing at various steps are indicated in red.

To test whether the cCadC‐Aβ42 strategy can be used to identify cyclic peptides that bind AβOs, we subjected *E. coli* harboring a ∼10^7^ member SICLOPPS cyclic peptide library to select for increased chloramphenicol resistance arising from cCadC‐dependent expression of chloramphenicol acetyltransferase (Figure 1a). In SICLOPPS, a precursor fusion consisting of the C‐terminal half of a *trans*‐splicing split intein, the peptide sequence to be cyclized, and the N‐terminal half of the same split intein undergoes splicing after ribosomal translation to produce head‐to‐tail cyclized peptides (Scheme 1). Mechanistically, the SICLOPPS precursor **1** first undergoes an initial *N*‐to‐*S* acyl shift followed by trans‐thioesterification to liberate the N‐terminal intein and generate lariat thioester intermediate **3**. Following this, cleavage by an asparagine residue in the C‐terminal intein produces the cyclic thioester species **4**, which undergoes a final *S*‐to‐*N* acyl shift to produce head‐to‐tail cyclic peptide **5**.

Our SICLOPPS library consisted of 7‐mer CP sequences where the first residue was fixed to cysteine (required for splicing) and the next six residues were randomized using NNK codons. We also used the Cfa GEP split intein (Cfa) in place of the Ssp intein that is typically employed in SICLOPPS due to Cfa's reported improved splicing kinetics [45]. Finally, we lowered the selection stringency by fusing cCadC to the ΔE22 mutant of Aβ42, which generates more AβOs and increases cCadC activation relative to wild‐type Aβ42 (Aβ42 WT) [43, 46].

Sequencing of eight surviving colonies after selection identified three distinct sequences: CFVQLFF (3/8 colonies sequenced), CRLISFF (4/8 colonies), and CGLLVFP (1/8 colonies), suggesting that the postselection population was highly converged. The CRLISFF sequence coincided with a mutation from proline to leucine at the 23^rd^ residue in the C‐terminal split intein. We then tested the activity of the hits against cCadC‐Aβ42 WT in a luminescence assay measuring cCadC activation of bacterial luciferase expression. CFVQLFF and CRLISFF (P23L) robustly activated cCadC‐Aβ42 transcription (Figure 1b and Figure S1a‐b). At higher expression levels of CFVQLFF and CRLISFF (P23L), we observed a decrease in luminescence signal, possibly due to cyclic peptide self‐association (Figure S1a‐b). In contrast, CGLLVFP failed to produce increased luciferase transcription, suggesting that it is a false positive (Figure S1c). Additionally, both CFVQLFF and CRLISFF (P23L) failed to yield luminescence when tested against the Aβ42 F20S/L35P monomeric mutant fusion, suggesting that the peptides do not force Aβ42 to artificially oligomerize (Figure 1b).

Next, we examined the effect of the P23L mutation. Reverting the mutation caused severe growth inhibition of cells expressing the CRLISFF SICLOPPS precursor and abolished P_*cadBA*_ transcription (Figure 1c, Figure S2), suggesting that P23L may have arisen due to its ability to mitigate CRLISFF toxicity. In contrast, installing P23L in the CFVQLFF SICLOPPS precursor ablated its ability to activate cCadC‐Aβ42 WT but had no observable effects on cell growth (Figure 1c). Because P23 was one of the mutations installed in Cfa to enhance its splicing efficiency, we examined its effect on splicing of the SICLOPPS precursor for both sequences. P23L decreased but did not abolish splicing for the SICLOPPS precursor for CRLISFF but interestingly had no effect on splicing for the SICLOPPS precursor for CFVQLFF (Figure S3). We also observed reduced expression of the L23 SICLOPPS precursor for CRLISFF, consistent with this construct's growth inhibitory effect. These results suggest that the P23L mutation may act to reduce the toxicity and increase the expression level of the SICLOPPS precursor and splicing products for CRLISFF. Additionally, the reduction in splicing of CRLISFF (P23L) could suggest that its SICLOPPS precursor or a splicing intermediate might be active in the cCadC selection.

Accordingly, we assessed the dependence of CFVQLFF and CRLISFF (P23L) activity on intein splicing by installing a T69A/H72A double mutation in the block B region of the N‐terminal Cfa intein (Cfa_*N*_) or a H24L/F26L double mutation in block F of the C‐terminal intein (Cfa_C_). Mutations at these positions have been used to assess whether selected SICLOPPS cyclic peptides require complete splicing for their activity [17, 42]. The Cfa_*N*_ T69A/H72A mutation impairs the initial N‐to‐S acyl shift and stalls splicing at SICLOPPS precursor **1** while the Cfa_C_ H24L/F26L mutation prevents asparagine cyclization and pauses splicing at lariat intermediate **3**. Both mutations greatly decreased the ability of CFVQLFF and CRLISFF (P23L) to activate cCadC‐Aβ42 WT relative to the parent Cfa intein (Figure 1d), initially suggesting to us that the activity of both sequences was strongly dependent on the intracellular accumulation of the fully spliced cyclic peptide product **5**.

**FIGURE 2:**
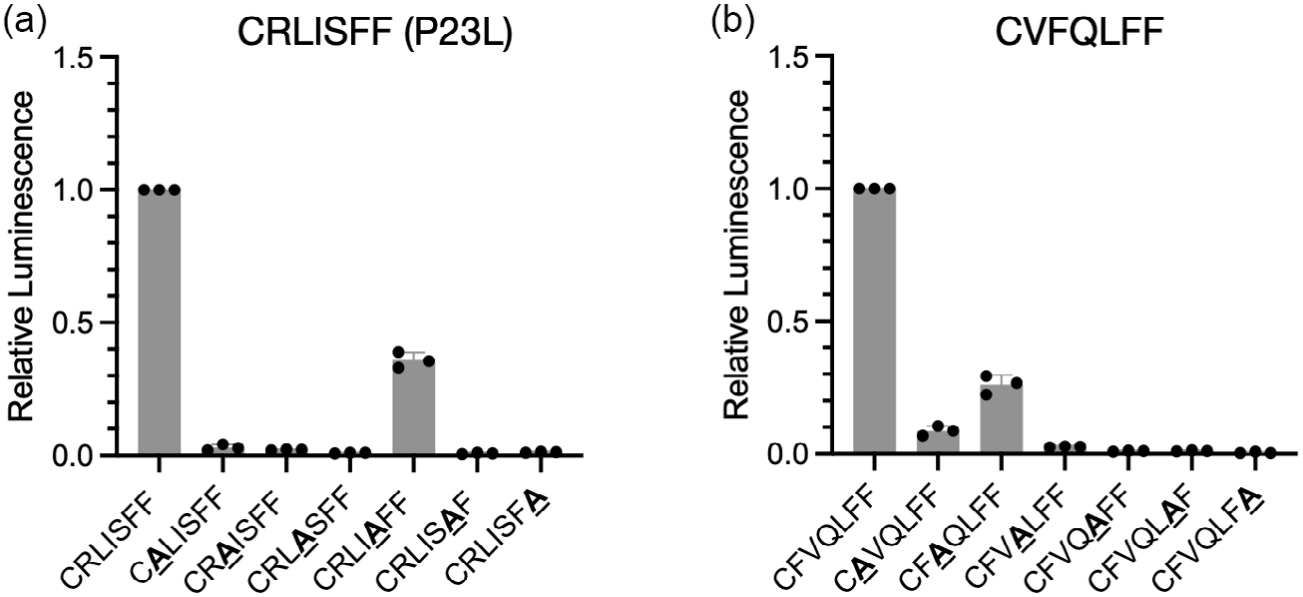
Alanine scanning of selected hit sequences. Effect of alanine substitutions in the (a) CRLISFF or (b) CFVQLFF sequence on *luxAB* transcriptional activation by cCadC‐Aβ42 WT.

Finally, we performed alanine scanning analysis of the selected sequences to determine the contribution of each amino acid to their activities. Replacement of any of the six selected residues with alanine in CFVQLFF or CRLISFF (P23L) greatly reduced cCadC‐Aβ42 WT activation (Figure 2). Only the valine in CFVQLFF and the serine in CRLISFF (P23L) displayed moderate tolerance of mutation to alanine, suggesting that all residues in these sequences contribute strongly to activity.

### 2.2 In Vitro Aβ42 Aggregation Activity of Cyclo‐CRLISFF

It is challenging to directly assess binding to Aβ42 oligomers generated from the aggregation cascade. However, a characteristic effect of AβO binders that activate cCadC‐Aβ42 is their ability to inhibit the secondary nucleation step of the Aβ42 aggregation cascade (Figure S4) [43]. Secondary nucleation inhibitors can be identified through assessing their impact on Aβ42 aggregation kinetics in in vitro ThT fluorescence assays as they primarily reduce the slope of aggregate formation [47]. Therefore, we determined whether our selected sequences inhibited Aβ42 aggregation in ThT assays, and if so, which step(s) of the aggregation mechanism they acted upon. For these experiments, we attempted to synthesize both *cyclo‐*CRLISFF and *cyclo*‐CFVQLFF. However, the linear CFVQLFF sequence proved to be insoluble in aqueous solution, suggesting that, postcyclization, it would likely be incompatible with the buffer conditions used for ThT assays. Therefore, we only evaluated *cyclo‐*CRLISFF in these experiments.

**FIGURE 3:**
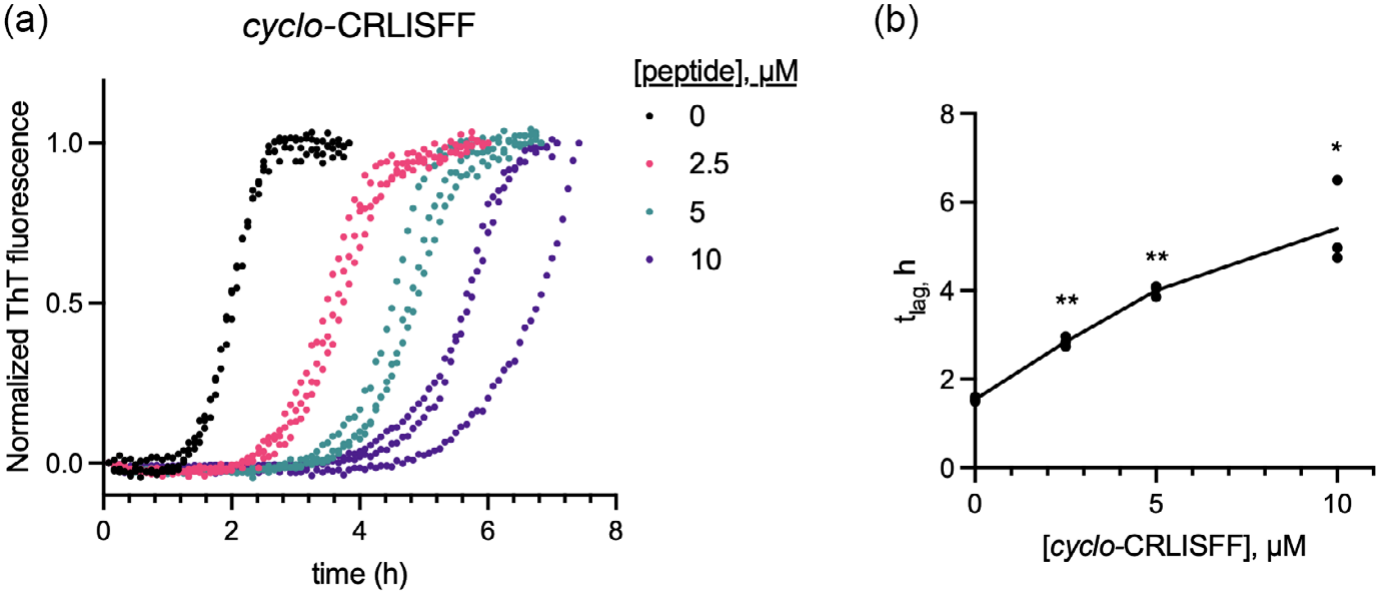
(a) In vitro ThT fluorescence assay of Aβ42 aggregation in presence of varying concentrations of *cyclo*‐CRLISFF. Assay conditions: 3 µM Aβ42 in 20 mM Sodium Phosphate, 0.2 mM EDTA, pH 8.0, 1% acetonitrile, 0.1% TWEEN‐20. Data reflects three technical replicates plotted as individual values. (b) Aggregation lag time (t_lag_) derived by fitting data shown in panel (a) * *P<=* 0.05, ** *P* <= 0.01 (Student's *t*‐test, 2‐tailed).

We first tested the activity of *cyclo*‐CRLISFF in ThT fluorescence assays using a highly reproducible protocol that enables interrogation of the microscopic steps of Aβ42 aggregation (Figure 3) [6]. Here, the buffer was supplemented with 1% acetonitrile and 0.1% TWEEN‐20, conditions previously reported to ensure cyclic peptides remain soluble and monomeric in ThT assays [16]. *Cyclo*‐CRLISFF strongly inhibited Aβ42 aggregation in vitro (Figure 3a). However, instead of inhibiting secondary nucleation, *cyclo*‐CRLISFF primarily delayed aggregation lag time (t_lag_) (Figure 3b). This indicated a potential discrepancy between the mechanism of the cCadC selection and the in vitro activity of the hit it identified.

We wondered if the discrepancy between the cCadC selection and these in vitro results might be due to acetonitrile and TWEEN‐20 perturbing the aggregation mechanism of Aβ42. We used AmyloFit [48] to perform global fitting analysis of Aβ42 aggregation in the presence of 1% acetonitrile and 0.1% TWEEN‐20 (Figure S5). The aggregation kinetics under these conditions no longer fit a secondary nucleation‐dominated model but instead agree with a model that also incorporates fragmentation, suggesting a change in the mechanism of Aβ42 aggregation. This convolutes our ability to assess the effect of *cyclo*‐CRLISFF on the microscopic steps of Aβ42 aggregation. Unfortunately, our efforts to detect *cyclo*‐CRLISFF activity in the absence of acetonitrile and TWEEN‐20 were unsuccessful (Figure S6), potentially due to insolubility or increased self‐association of *cyclo*‐CRLISFF.

### 2.3 Selected Sequences are Active as Intein‐Bound Intermediates

The lack of secondary nucleation inhibition by *cyclo*‐CRLISFF in in vitro assays raised the possibility that the active species in the cCadC selection may not be the fully spliced SICLOPPS product **5**. Although our initial experiments appeared to rule out strong activity from the SICLOPPS precursor **1** or lariat intermediate **3** (Figure 1d), we elected to examine additional mutations: Cfa_*N*_ C1A, which should also prevent the initial *N*‐to‐*S* acyl shift to stop at precursor **1**, and Cfa_C_ N36A, which should prevent Asn cyclization to stop at intermediate **3**. We also examined thioester intermediate **2**, which is formed by the initial *N*‐to*‐S* acyl shift. Pausing splicing at this step requires mutation of the first cysteine of the cyclic peptide sequence itself into an alanine (extein C1A).

**FIGURE 4:**
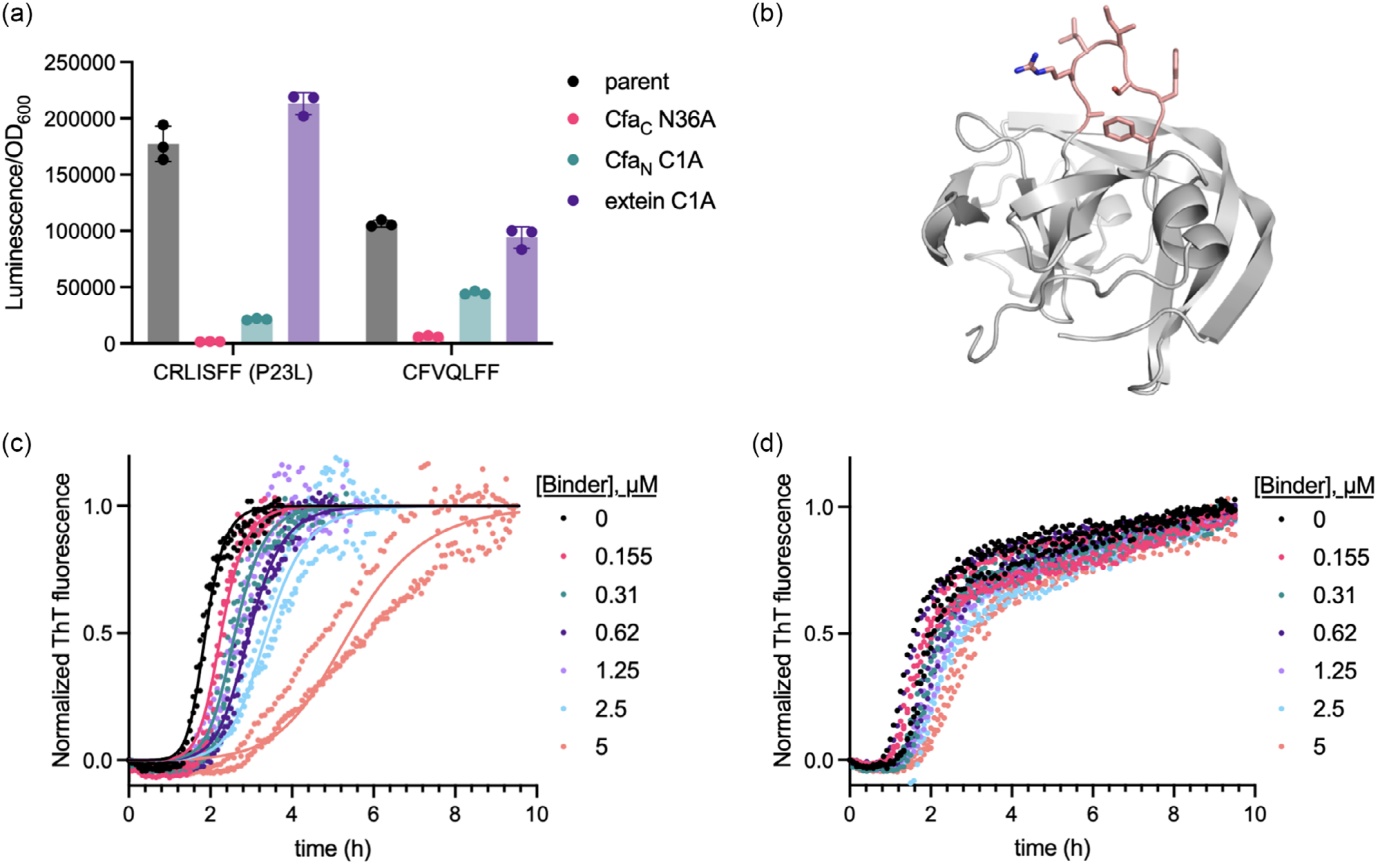
Activity of intein‐bound SICLOPPS precursors. (a) Effect of additional intein mutations on the activities of CRLISFF (P23L) and CFVQLFF tested on cCadC‐Aβ42 WT. (b) AlphaFold [49] model of Cfa intein with the ARLISFF sequence shown as pink sticks. (c) In vitro ThT fluorescence assay of Aβ42 aggregation in presence of varying concentrations of Cfa P23L‐ARLISFF. (d) In vitro ThT fluorescence assay of Aβ42 aggregation in presence of varying concentrations of Cfa P23L‐ARLISFA. (c‐d) Assay conditions: 3 µM Aβ42 in 20 mM Sodium Phosphate, 0.2 mM EDTA, pH 8.0, Data reflects three technical replicates plotted as individual values. Lines in panel (c) represent fits to a selective reduction of the secondary nucleation rate constant using Amylofit [48].

While the Cfa_C_ N36A mutation severely reduced activity from both sequences, the Cfa_*N*_ C1A mutant was modestly active (Figure 4a). In comparison, the Cfa_*N*_ H72A/T75A mutation we initially tested should have also stalled splicing at the same species (precursor **1**) yet was completely inactive. This discrepancy between the activities of the Cfa_*N*_ H72A/T75A and Cfa_*N*_ C1A mutants indicates that, although they are both presumably paused at the first step of *trans‐*splicing, there exists some difference between these mutants, such as the conformation or exposure of the intein‐bound peptide sequence. Additionally, to our surprise, the extein C1A mutants of both sequences strongly activated cCadC transcription, on par with the unmutated parent sequence (Figure 4a). These results suggest that both sequences may retain activity as either precursor **1** and/or the intein‐bound thioester **2** formed immediately after the first *N‐*to‐*S* acyl shift of the SICLOPPS splicing mechanism but lose activity if trapped at other splicing intermediates. An AlphaFold prediction of the structure of Cfa intein‐bound ARLISFF suggests that this sequence may be displayed in a loop‐like fashion (Figure 4b); however, the exact positions of the ARLISFF residues are uncertain (Figure S7).

To validate the results from the above experiment, we purified the extein C1A mutant of CRLISFF, Cfa(P23L)‐ARLISFF, and tested it in a ThT assay of Aβ42 aggregation without the addition of acetonitrile or TWEEN‐20. Under these conditions, Cfa(P23L)‐ARLISFF delayed Aβ42 aggregation by primarily inhibiting secondary nucleation (Figure 4c and Figure S8), consistent with the mechanism of oligomer binders we have previously identified using the cCadC selection [43]. As a control, we also purified and tested the mutant Cfa(P23L)‐ARLISF **A** , which contains a F to A mutation that inactivated Cfa(P23L)‐CRLISFF in the cCadC assay (Figure 2a). Cfa(P23L)‐ARLISFA showed greatly diminished activity in ThT assays (Figure 4d), indicating that the activity we observed from Cfa(P23L)‐ARLISFF did not stem from the Cfa(P23L) intein scaffold. These results suggest that Cfa(P23L)‐ARLISFF binds cCadC‐Aβ42 oligomers and that this activity translates into selective secondary nucleation inhibition in ThT assays.

### 2.4 Peptide Sequences Retain Aβ42 Activity when Grafted onto a Different Protein Scaffold

Macrocyclic peptides identified through mRNA display have been demonstrated to retain biological activity when grafted into a protein scaffold. This type of peptide grafting can offer advantages such as increasing the stability and ease of production (through expression) of the resulting chimeric construct while retaining the binding activity of the parent cyclic peptide [50]. Because we observed the CRLISFF sequence to be active in both a head‐to‐tail cyclic peptide and a protein‐bound context, we wondered if it might similarly retain a binding‐competent conformation when grafted into a loop of an unrelated proteinogenic scaffold. This would also create a more stable AβO binder, as the intein‐bound peptide was susceptible to hydrolysis of the thioester (Figure S9).

**FIGURE 5:**
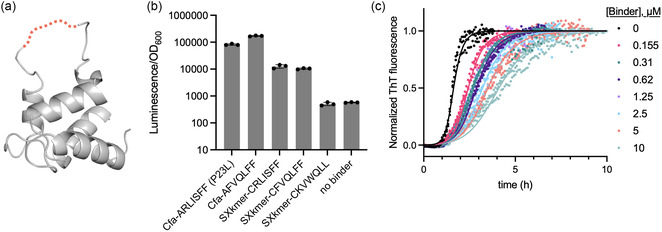
Characterization of the peptide sequence and the intein on AβO binding activity. (a) AlphaFold [49] model of Sxkmer scaffold (gray) with the grafted loop shown as a dashed orange line. (b) Effect of CRLISFF and CFVQLFF sequences grafted onto the Sxkmer scaffold on *luxAB* transcriptional activation by cCadC‐Aβ42 WT. The activities of extein C1A SICLOPPS precursors are shown in comparison, as well as negative control binder (SXkmer‐CKVWQLL) and a no binder control. To better resolve differences in activity in this assay, the data are plotted in log scale. (c) in vitro ThT fluorescence assay of Aβ42 aggregation in the presence of varying concentrations of Sxkmer‐CRLISFF. The data was fit to a selective reduction of secondary nucleation constants in Amylofit. Assay conditions: 3 µM Aβ42 in 20 mM Sodium Phosphate, 0.2 mM EDTA, pH 8.0. Data reflects three technical replicates plotted as individual values. Lines represent a fit to a selective reduction of the secondary nucleation rate constant using Amylofit [48].

To this end, we placed the CRLISFF and CFVQLFF sequences into an SXkmer loop (Figure 5a). SXkmers are binders engineered from the S100G calcium binding protein that are highly stable, monomeric, and have been previously used to engineer Aβ42 inhibitors [47]. Both CRLISFF and CFVQLFF retained activity on cCadC‐Aβ42 oligomers when introduced into the SXkmer loop, albeit at lower levels compared to the Cfa intein‐bound intermediates (Figure 5b). In contrast, CKVWQLL, a sequence identified from a SICLOPPS selection for inhibiting Aβ42‐GFP aggregation (as opposed to AβO binding) [16], failed to yield cCadC signal when grafted (Figure 5b). The observed lack of activity from the CKVWQLL sequence was not due to differences in expression, as this SXkmer graft was found to express abundantly (Figure S10). The activity of the CRLISFF SXkmer graft also translated into to inhibition in vitro: purified SXkmer‐CRLISFF delayed Aβ42 aggregation in ThT fluorescence assays, acting primarily through selective reduction of Aβ42 sary nucleation (Figure 5c and Figure S11), consistent with an oligomer binding mechanism. Thus, sequences identified from our SICLOPPS selections can retain both their overall activity and mode of Aβ42 inhibition when grafted onto a different protein.

## 3 Conclusion

We selected a 7‐mer SICLOPPS cyclic peptide library for Aβ42 oligomer binders, using a cCadC‐based cellular reporter for Aβ oligomer binding [43]. We identified two sequences, CRLISFF and CFVQLFF, which are quite distinct from SICLOPPS 7‐mer sequences enriched in a previous screen of for Aβ42‐GFP aggregation inhibitors, consistent with differences in mechanism between the cCadC‐Aβ42 and Aβ42‐GFP reporters [16]. Based on testing with well‐precedented intein mutations, both CRLISFF and CFVQLFF initially appeared to be dependent on SICLOPPS intein splicing for activity. Additionally, chemically resynthesized *cyclo*‐CRLISFF robustly inhibited Aβ42 aggregation when tested in vitro. To our surprise, the mechanism of Aβ42 inhibition by *cyclo*‐CRLISFF (delay of aggregation onset) was more consistent with binding of monomeric rather than oligomeric Aβ42 species. A potential cause for this is that our ThT buffer conditions, which included acetonitrile and TWEEN‐20 to reduce cyclic peptide self‐association, altered the mechanism of Aβ42 aggregation. However, we cannot conclusively say that this alone is responsible for the inconsistency of *cyclo*‐CRLISFF's behavior, and it remains unclear if *cyclo*‐CRLISFF acts through binding Aβ42 oligomers.

The discrepancies observed in CRLISFF activity between the cellular cCadC assay and in vitro led us to more thoroughly examine the potential for SICLOPPS splicing intermediates to exhibit activity by testing additional mutants to pause intein splicing at various steps. We found that two different sets of mutations (T69A/H72A and Cfa_*N*_ C1A), both predicted to stall splicing at SICLOPPS precursor **1**, had divergent effects in the cCadC assay. While T69A/H72A mutants were inactive, Cfa_*N*_ C1A mutants of both CRLISFF and CFVQLFF showed appreciable activity, suggesting conformational differences between these mutants may influence productive display of the peptide sequence for AβO binding; alternatively, these mutants may not accumulate chemically identical intermediates. Furthermore, we found that extein C1A mutants, which pause splicing at thioester intermediate **2**, exhibited comparable activity to the parent SICLOPPS construct. When purified and tested in vitro, the extein C1A mutant Cfa(P23L)‐ARLISFF inhibited Aβ42 by principally delaying secondary nucleation, consistent with an oligomer binding mechanism. These results suggest that intermediates prior to the *trans‐*thioesterification step of SICLOPPS splicing can be active in our cCadC selection. When using SICLOPPS to generate cyclic peptides, all possible splicing intermediates are present in cells and subject to selection, with the nature of the selection, population of different intermediates, and the context in which the peptide sequence is displayed all likely factoring into whether a specific intermediate is active. However, most studies employing SICLOPPS examine only a subset of these intermediates. Our findings suggest that it may be necessary to examine a wider set of splicing mutants to fully elucidate the active specie(s).

We also found that the CRLISFF sequence is active when grafted into an engineered S100G protein both in the cCadC assay and in vitro. In the latter case, we also observed preservation of a secondary nucleation inhibition mechanism. These experiments show that the CRLISFF sequence can inhibit Aβ42 aggregation in three very different forms (head‐to‐tail cyclized peptide, intein‐bound intermediate, and transplanted into a different protein). Our results are consistent with reports that macrocycles isolated from mRNA display are also active when inserted into different protein scaffolds [50] and with reports that sequences identified from SICLOPPS selections exhibit activity when grafted into a cyclotide [51]. Additionally, although we were unable to resolve the exact Aβ42 species *cyclo‐*CRLISFF recognizes, our study identifies both Cfa(P23L)‐ARLISFF and SXkmer‐CRLISFF as Aβ42 oligomer binders.

Several limitations of this study suggest directions for future work. First, we used the Aβ42 ΔE22 mutant in place of the wild‐type sequence in our selection to reduce stringency. Although our downstream experiments show that identified hits were active on wild‐type Aβ42, it is possible that use of Aβ42 ΔE22 may have influenced the sequences that initially enriched, and it may be interesting to reexamine this selection employing wild‐type Aβ42 as the target. Second, the hydrophobicity of the sequences enriched from our selections complicated downstream analysis. CFVQLFF was not tested due to insolubility in water, while *cyclo*‐CRLISFF showed no activity in the absence of acetonitrile and TWEEN‐20, suggesting inactivation by self‐association. Moving forward, more careful design of cyclic peptide libraries to include charged residues may improve solubility and reduce self‐association of selected sequences. Third, while the CFVQLFF sequence was active both as a SICLOPPS construct and an SXkmer graft in the cCadC assay, we did not test it in vitro, which caveats the generalizability of our study's observations. Finally, as our experiments were performed in artificial models of Aβ42 aggregation (*E. coli* and in vitro), testing in more disease‐relevant models of Aβ oligomer toxicity is required to assess the utility of our selected sequences in the broader context of AD.

## Supporting Information

Additional supporting information can be found online in the Supporting Information section.

## Funding

This work was supported by National Institute on Aging (Grant R21AG088715).

## Conflicts of Interest

The authors declare no conflicts of interest.

## Supporting information

Supplementary Material

## Data Availability

The data that support the findings of this study are available from the corresponding author upon reasonable request.
