# Supplementary material for: Identification of Secondary Nucleation Inhibitors of Amyloid‐β Aggregation by Cellular Selection of a SICLOPPS Library
Source: Chembiochem. 2026 Apr 27;27(8):e202500908. doi: 10.1002/cbic.202500908 (PMC13113203; doi:10.1002/cbic.202500908)
Supplement: Supplementary file 1 — Supplementary Material [file CBIC-27-e202500908-s001.pdf]

Supplemental Information

Materials and Methods

General Methods

Antibiotics (Gold Biotechnology) were used at the following working concentrations: chloramphenicol, 25 µg/mL, spectinomycin, 100 µg/mL, ampicillin, 50 µg/mL, 50 µg/mL kanamycin,10 µg/mL tetracycline, 50 µg/mL streptomycin. Inducers were made from stock concentrations: 100 µg/mL anhydrotetracycline (aTc), 1 M Isopropyl β-D-1-thiogalactopyranoside (IPTG). Nuclease Free water (Omega-Biotek) was used for PCR reactions and cloning. For all other experiments, water was purified using a MilliQ purification system (Millipore).

Q5U DNA polymerase or Q5 DNA polymerase (New England Biolabs) was used to generate PCR products for plasmid construction. A full list of plasmids used in this work is given in **Supplementary Table 1**. Data were plotted using GraphPad Prism 10.2.2.

Bacterial strains, culture conditions and media

E. coli strain S2060 was used for bacterial luminescence assays and general plasmid cloning. S2060 strain is derived from strain DH10β with the flowing genotype: *endA1 recA1 galE15 galK16 nupG rpsL ΔlacIZYA araD139 Δ(ara,leu)7697 mcrA Δ(mrr-hsdRMS-mcrBC) proBA::pir116 araE201 ΔrpoZ Δflu ΔcsgABCDEFG ΔpgaC λ– F′ proA + B + Δ(lacIZY) zzf::Tn10 lacIQ1 PN25-tetR luxCDE Ppsp(AR2) lacZ luxR Plux groESL*. S2060 *ΔcadCBA* was used to select cyclic peptide sequences to reduce background P_{cadBA} transcription from endogenous CadC as previously reported.[43] BL21 DE3 pLysS cells were used for Aβ42 purification. NEB 10β cells were used to clone the SICLOPPS library. Bacterial cell culture maintenance and plasmid transformation were performed as previously described.[43]

Bacterial luminescence assays

Overnight cultures of cells harboring the plasmids of interest were generated by inoculating single colonies in 1 mL of 2xYT medium harboring maintenance antibiotics while shaking at 37°C. Saturated overnight cultures were diluted 1:100 into 1 mL of Davis Rich Media containing antibiotics and shaken until cultures reached optical density at 600 nm absorbance (OD_{600}) of 0.4 at 37°C. Then, 10 µL of 10 mM IPTG and 200 ng/mL aTc were added to the medium to induce production of SICLOPPS constructs and cCadC-Aβ42 variants. 3 hours post induction, 100 µL of cells were transferred to black 96-well clear-bottom plates. Luminescence and OD_{600} were measured using a Tecan Infinite Mplex plate reader using Tecan i-Control 2.0 software. OD normalized luminescence values were generated by dividing raw luminescence values by OD_{600} values subtracted by background OD_{600} of black 96 well clear-bottom plates.

Cfa SICLOPPS library generation

The Cfa SICLOPPS library was generated by USER cloning as previously described with a few modifications.[43] Briefly, primers BL0674 and BL0671 were used to amplify pBL141Z. The PCR product was DpnI digested for 1 h to remove template DNA and gel purified. 0.55 pmol of DNA was assembled via USER assembly with the addition of T7 DNA ligase for 37°C for 2 h (5 µL USER, 25 µL T7 DNA ligase buffer, 2.5 µL T7 DNA ligase, 17.5 µL purified DNA). The resulting mixture was subjected to PCR purification and 1 µL of 180 ng/mL DNA was transformed into electrocompetent NEB10β cells (~10^9 cells/mL). Cells were then recovered in 2xYT media supplemented with 20 mM glucose and shaken at 37°C for 1 h. An aliquot was taken to estimate transformation efficiency by dilution plating and colony counting. An additional 19 volumes of 2xYT were added to the remaining culture, which was allowed to grow overnight at 30°C with shaking. The library DNA was then extracted by midiprep.

Cfa SICLOPPS library selection

Electrocompetent selection cells (S2060 *ΔcadCBA* cells harboring plasmids pTW390B (P_{tet} cCadC–Aβ42 ΔE22) and pBL140 (P_{cadBA} *cat*)) were transformed with the Cfa SICLOPPS library. Cells were recovered for 1 h in outgrowth media (2xYT supplemented with 20 mM glucose), then induced with 200 ng/mL aTc (induces cCadC-Aβ42) and 0.1 mM IPTG (induces Cfa SICLOPPS library members) and allowed to grow for another 2 h. A total of 10^7 transformants were then plated on LB agar containing inducers (200 ng/mL aTc and 0.1 mM IPTG), maintenance antibiotics, and varying levels of chloramphenicol (40 µg/mL and 50 µg/mL). The plates were then incubated at 37°C for ~1.5 days at which point the surviving colonies were scraped and plasmids extracted by mini prep. The surviving library sequences were then amplified by PCR and subcloned into the pBL141Z backbone by USER assembly. The subcloned library was subjected to a second round of selection (50 µg/mL chlor) by using the same procedure described above. Surviving colonies were analyzed by Sanger sequencing.

Analysis of Cfa (P23 or L23) CRLISFF or CFVQLFF by SDS-PAGE

Saturated overnight cultures harboring the plasmids of the Cfa variants were diluted 1:100 into 2xYT media containing maintenance antibiotics. Cells were grown until OD_{600} reached 0.6, at which point 0.1 mM IPTG was added to induce expression of the Cfa variants. Induced cultures were then shaken overnight at 37°C. 1 mL of culture at OD_{600} of 1.8 was spun down. For the total cell fraction, the cell pellet was resuspended in 70 µL H_2O. Then, 10 µL of the cell resuspension was added to 10 µL 4X Laemmli sample buffer supplemented with 100 mM DTT and 20 µL of H_2O and boiled at 98 °C for 10 min. For the soluble fraction, the cell pellet was resuspended in lysis buffer (B-PER (ThermoFisher 78243)) supplemented with 10 mM $MgCl_2$, 1 µg/µL lysozyme, and 1 mM phenylmethylsulfonyl fluoride (PMSF, RPI research products). The samples were incubated on ice for 20 min and spun down at 20,000 g at 4 °C for 25 min. 10 µL of the resulting supernatant was added to 10 µL 4X Laemmli sample buffer supplemented with

100 mM DTT and 20 µL of H_2O and boiled at 98 °C for 10 min. Proteins were separated using a pre-cast 4-20% Tris-glycine polyacrylamide gel (Bio-Rad) at 220 V for 30 mins in SDS-PAGE buffer (25 mM Tris, 190 mM glycine, 0.1% SDS, pH 8.3).

Expression and purification of Cfa(P23L)-ARLISFF, Cfa(P23L)-ARLISFA, and SXkmer-CRLISFF

Purification of binders was performed as previously described with few modifications.[43] Briefly, overnight cultures of BL21 DE3 cells that had overexpressed binders was harvested by centrifugation. The cell pellets were resuspended in lysis buffer (10 mM Tris-HCl, 0.1 mM EDTA, pH 7.5) and sonicated to extract the soluble fraction. The lysate was spun down by centrifugation at 18,000g for 1 h. Proteins was isolated using affinity chromatography with nickel Sepharose resin (Cytiva) and purified using SEC on a Superdex 200 16/600 column with the BioRad NGC Quest 10 Plus system. Pure fractions were flash-frozen in liquid nitrogen and stored at -80 °C.

ThT fluorescence assays

A 1 mM stock of ThT (ChemCruz) solution was prepared in ThT buffer (20 mM sodium phosphate and 0.2 mM EDTA, pH 8.0) by first sonicating to dissolve ThT then filtering through a 0.22 µm filter to remove particulates.

Cyclo-CRLISFF was dissolved at 1 mM in 50% acetonitrile/H_2O to generate a stock solution. From this stock solution, *cyclo*-CRLISFF solutions at 2X the desired final concentrations were prepared in ThT buffer supplemented with 2% acetonitrile and 0.2% TWEEN-20 or 2% DMSO. Then, 40 µL of prepared *cyclo*-CRLISFF solutions were transferred into a 96-well black-walled, half-area clear-bottom, nonbinding surface plate (Corning 3881). For other binders, binder solutions at 2X the desired final concentrations were prepared in ThT buffer on ice in low protein binding microcentrifuge tubes. Then, 40 µL of binder solution was

transferred into a 96-well black-walled, half-area clear-bottom, nonbinding surface plate (Corning 3881).

Aβ42 purification from inclusion bodies was performed as previously described.[43] Lyophilized Aβ42 was dissolved in 6 M guanidine hydrochloride and purified by SEC using a Superdex 75 10/300 column to obtain highly monomeric starting material. Aβ42 concentration was estimated using 280 nm absorbance of the center of the monomer peak assuming a molar absorptivity of 1,440 M^{-1} cm^{-1}. A 2X master mix containing 6 μM of monomeric Aβ42 and 20 μM ThT was prepared in ThT buffer. 40 μL of the Aβ42/ThT master mix was added to the 40 μL *cyclo*-CRLISFF (or other binder) solutions prepared in the 96 well plate. The plate was immediately placed into a Tecan Infinite Mplex plate reader and incubated at 37°C without shaking for 14–18 h. ThT fluorescence values were read every 5 mins using 440 nm excitation and 480 nm emission. Raw ThT fluorescence curves were analyzed using the Amylofit 2.0 software.[47]

<u>*Solid-phase peptide synthesis.*</u>

<u>Abbreviations</u>

Dbz = 3,4-diaminobenzoic acid

DCM = dichloromethane

DIPEA = diisopropylethylamine

DMF = dimethylformamide

Fmoc = 9-fluorenylmethyloxycarbonyl

HATU = 1-[Bis(dimethylamino)methylene]-1H-1,2,3-triazolo[4,5-b]pyridinium 3-oxide hexafluorophosphate

HPLC = high-performance liquid chromatography

MeCN = acetonitrile

Nbz = N-acylbenzimidazolinone

TFA = trifluoroacetic acid

TIPS = triisopropylsilane

cyclo-CRLISFF

Fmoc-Dawson-Dbz resin (100 mg) was added to solid-phase extraction cartridge with preinserted frit (Agilent, 6 mL capacity) and swelled in 2 mL dichloromethane with agitation by rotation for 40 minutes. The DCM was drained and the resin washed three times with ~2 mL DCM followed by ~2 mL DMF. The resin was deprotected with 2 mL of 20% piperidine in DMF with rotation for 15 minutes. The 20% piperidine solution was drained and the resin washed with ~2 mL DMF three times. Subsequent couplings were achieved using 4 equivalents Fmoc-protected amino acid, 3.9 equivalents HATU, and 8 equivalents DIPEA dissolved in 2 mL DMF. Each solution was added to the resin and rotated for at least 1 hour. Between each coupling, the solution was drained from the cartridge and the resin was washed three times with ~2 mL DMF before the resin was rotated in 2 mL 20% piperidine in DMF solution for 15 minutes. Following treatment with 20% piperidine the resin was washed three times with ~2 mL DMF before proceeding to the next coupling. The final cysteine coupling was performed with Boc-Cys(Trt)-OH. Following the final Cys coupling, the resin was washed three times with DMF, then three times with DCM.

Following peptide couplings, the Dbz linker was activated to Nbz linker by adding a solution of 4 eq. 4-nitrophenyl chloroformate in 2 mL DCM to the resin and rotation for 30 minutes. The resin was washed three times with ~2 mL DCM. These steps were repeated once. The resin was then washed three times with ~2 mL DMF. Then, a solution of 110 µL DIPEA in 2 mL DCM was added to the resin followed by 10 minutes rotation. The solution was then drained from the cartridge. These steps were repeated twice more. The resin was washed three times

with ~2 mL DMF followed by washing three times with ~2 mL DCM, then finally washing three times with ~2 mL diethyl ether and allowed to dry.

The dry resin was transferred to a 5 mL round bottom flask where it was stirred in 2 mL solution of 95% TFA, 5% H_2O, and 5% TIPS for 2 hours. This cleavage mixture was passed through a cotton-plugged Pasteur pipette into -78ºC diethyl ether. The diethyl ether mixture was centrifuged for 20 minutes at 3000 RPM at 4ºC. The diethyl ether was decanted and the peptide pellet was dried before purification by HPLC using a 20-60% gradient of acetonitrile in H_2O. Fractions were analyzed with MALDI, combined, and lyophilized.

The purified linear peptide was cyclized in a solution of 0.1 M Na_2HPO_4, 6 M guanidinium hydrochloride, and 20% v/v acetonitrile in H_2O, pH 6.8–7.2, where the peptide was at 1 mM concentration. The solution was rotated in a 50ºC incubator for 2 hours. The cyclization mixture was reduced by adding 5 eq. of TCEP from a 200 mM aqueous TCEP solution. The solution was rotated at room temperature for 15 minutes before proceeding to purification via HPLC. The fractions were analyzed by MALDI, combined, and lyophilized to give 2.2 mg of final cyclized product (5.5% yield).

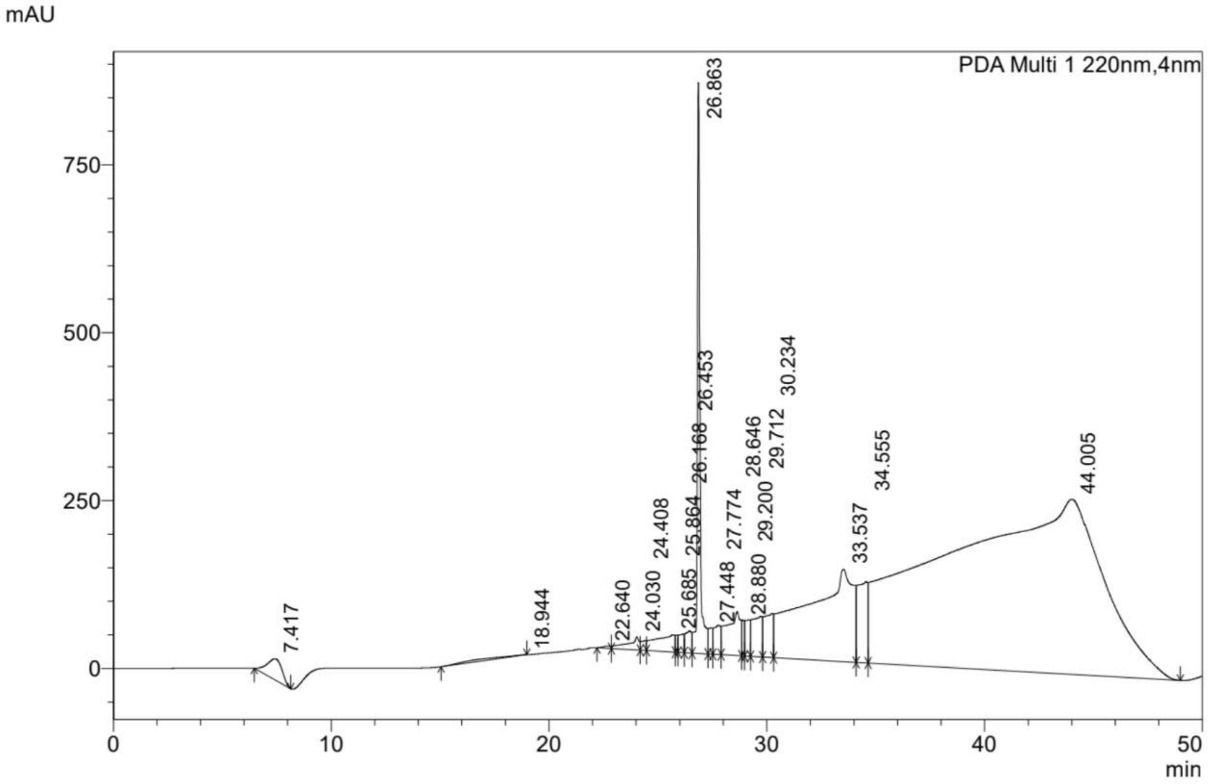

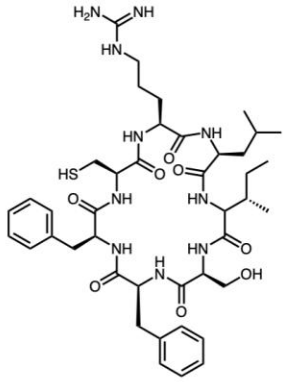

cyclo-CRLISFF
calculated $[M+H]^+$, 866.4473
found $[M+H]^+$, 867.4539

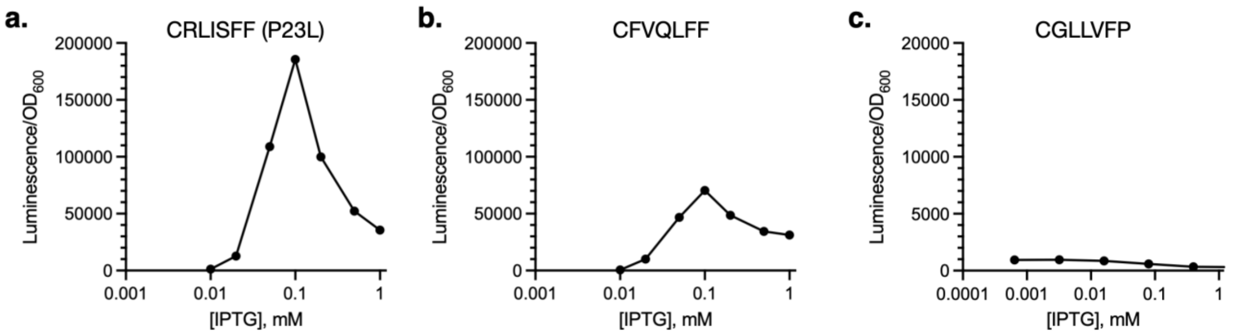

Supplementary Figure 1. cCadC-Aβ42 activation by selected sequences. Expression of SICLOPPS precursors for **(a)** CRLISFF (P23L), **(b)** CFVQLFF, and **(c)** CGLLVFP from the *tac* promoter was induced by addition of IPTG.

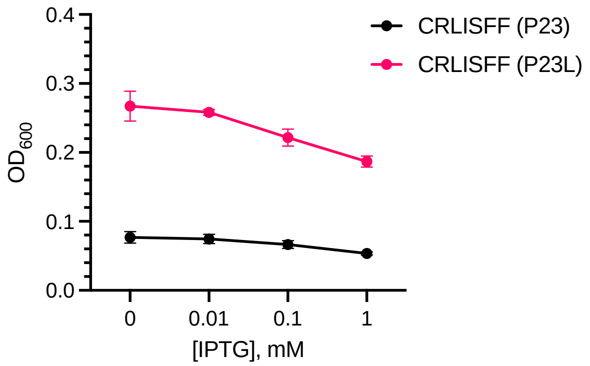

Supplementary Figure 2. Effect of P23L mutation on CRLISFF toxicity. Y-axis shows cell density measured as OD_{600}. SICLOPPS constructs were induced using IPTG from the *tac* promoter.

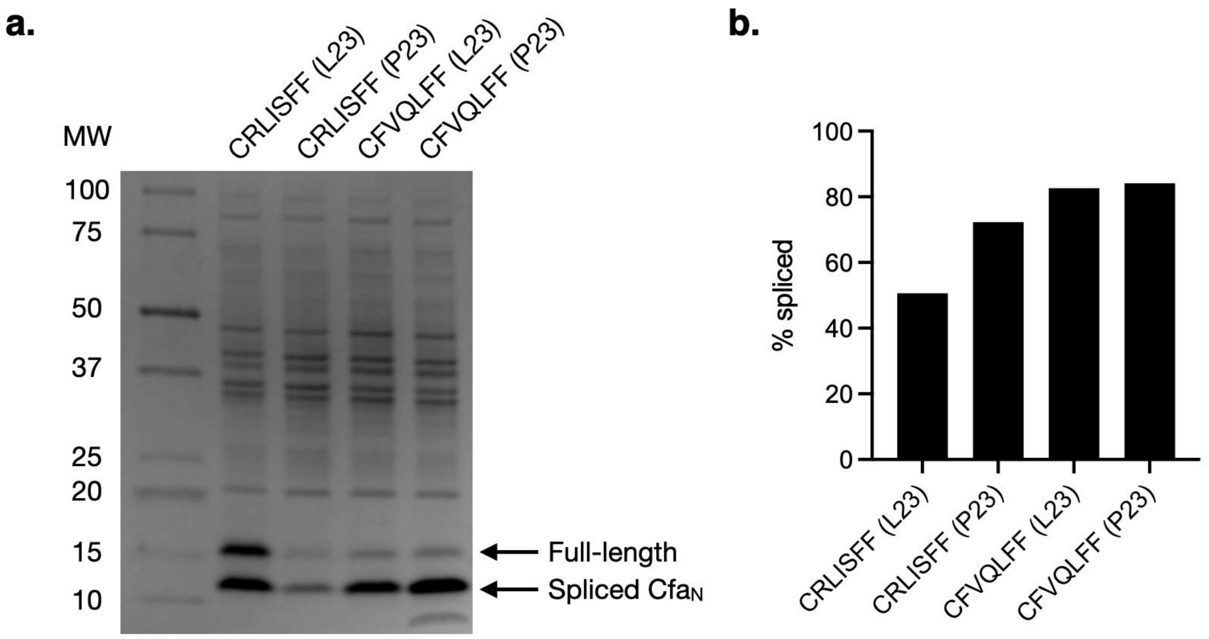

Supplementary Figure 3. Effect of P23L mutation on SICLOPPS precursor splicing. **(a)** SDS-PAGE of cell lysates expressing SICLOPPS precursors. Soluble protein fractions were separated on a 4-20% pre-cast gel. Proteins were detected by Coomassie staining. **(b)** Extent of splicing as measured by densitometry of the gel shown in panel **(a)**.

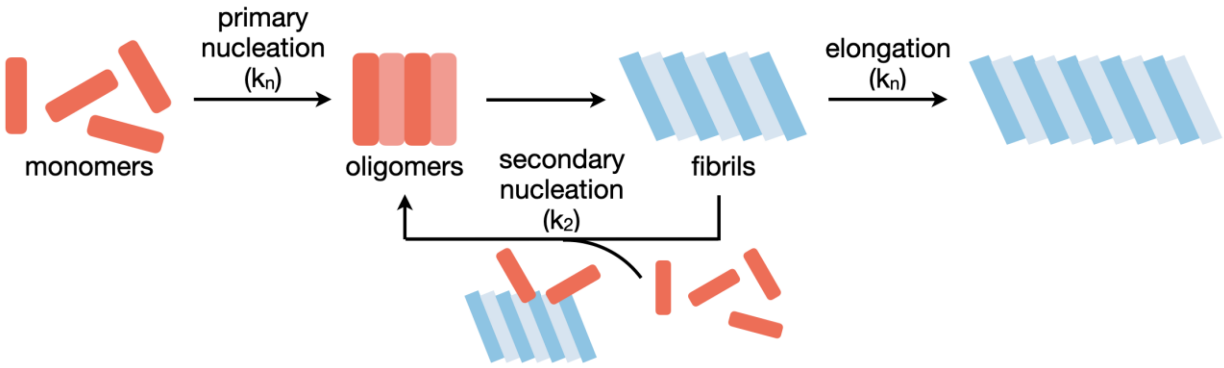

Supplementary Figure 4. Aggregation cascade of Aβ42 depicting the contribution of the individual microscopic steps (primary nucleation, elongation, and secondary nucleation).

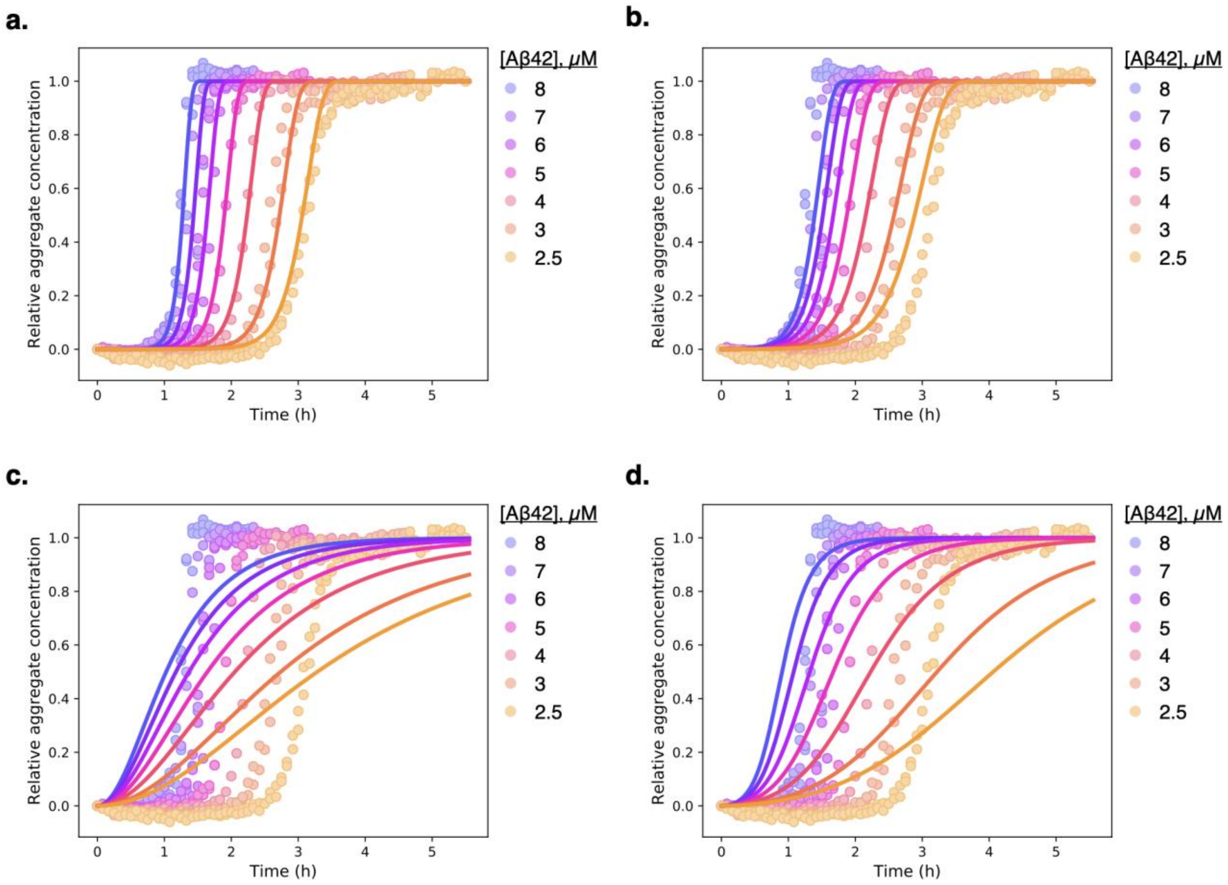

Supplementary Figure 5. Effect of acetonitrile and TWEEN-20 addition on Aβ42 aggregation kinetics. (a-d) Global fitting analysis of Aβ42 aggregation in ThT fluorescence assays in the presence of 1% acetonitrile and 0.1% TWEEN-20. Data was fitted using

Amylofit[47] to models incorporating **(a)** fragmentation and secondary nucleation, **(b)** a fragmentation dominated mechanism, **(c)** nucleation and elongation, and **(d)** a secondary nucleation dominated mechanism. Data reflects three technical replicates plotted as individual values.

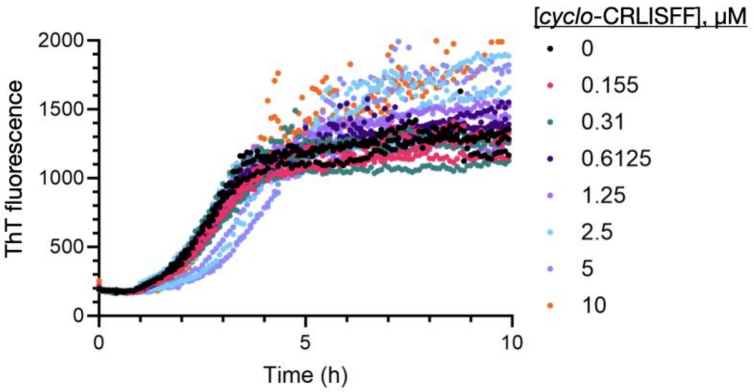

Supplementary Figure 6. *In vitro* ThT fluorescence assay of Aβ42 aggregation in presence of *cyclo*-CRLISFF without added acetonitrile and 0.1% TWEEN-20. Assay conditions: 3 μM Aβ42 in 20 mM Sodium Phosphate, 0.2 mM EDTA, pH 8.0, 1% DMSO. Data reflects three technical replicates plotted as individual values.

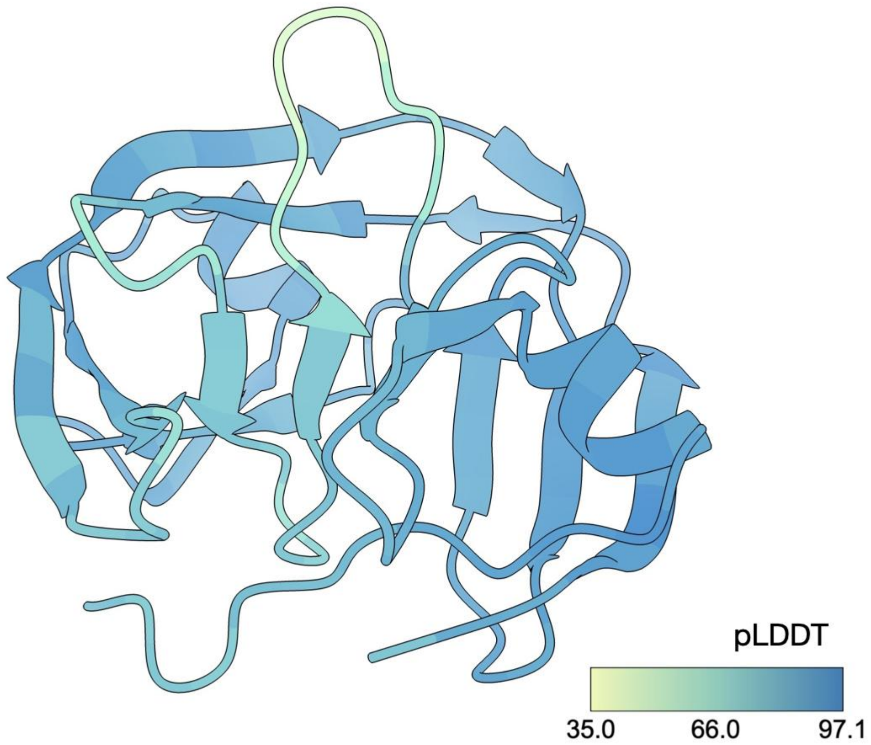

Supplementary Figure 7. AlphaFold structure of Cfa intein with the ARLISFF sequence with pLDDT confidence scores mapped.

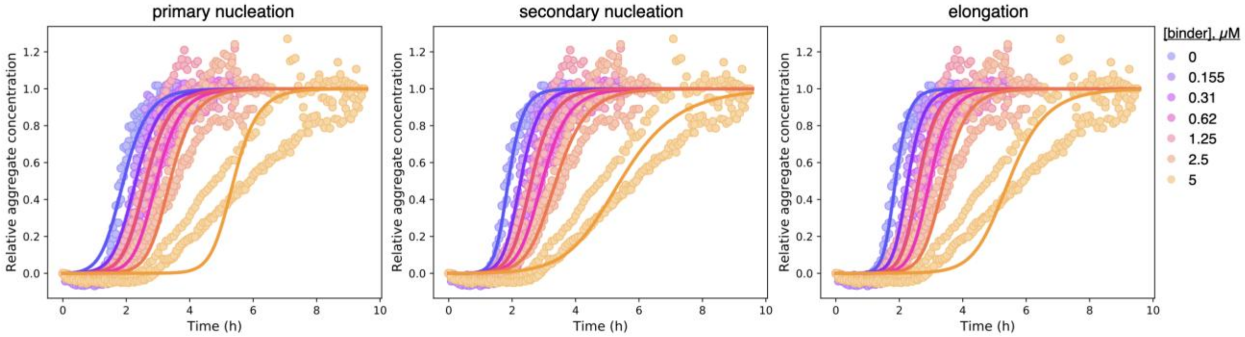

Supplementary Figure 8. ThT fluorescence assays of Aβ42 aggregation in presence of varying concentrations of Cfa(P23L)-ARLISFF fit to a selective reduction of primary nucleation, secondary nucleation, or elongation rate constants using Amylofit.[47]

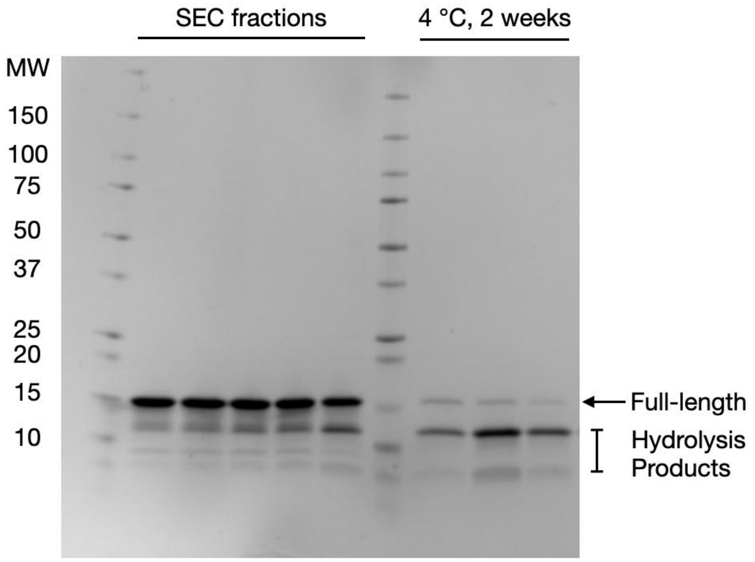

Supplementary Figure 9. SDS-PAGE of Cfa(P23L)-ARLISFF isolated directly after purification (SEC fractions) or stored at 4 °C for 2 weeks.

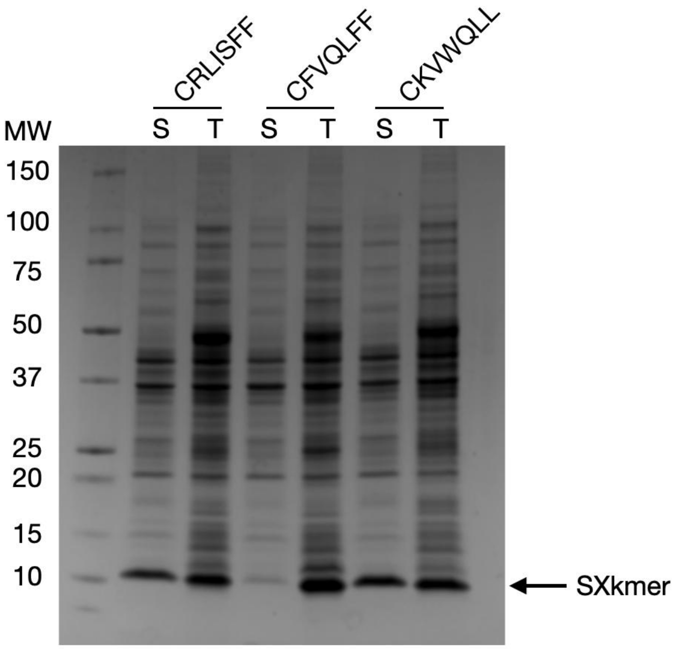

Supplementary Figure 10. SDS-PAGE of soluble (S) or total (T) protein extracts from cells expressing SXkmers grafted with loop sequences CRLISFF, CFVQLFF, and CKVWLL. The band corresponding to the SXkmer (MW ~10 kDa) is indicated.

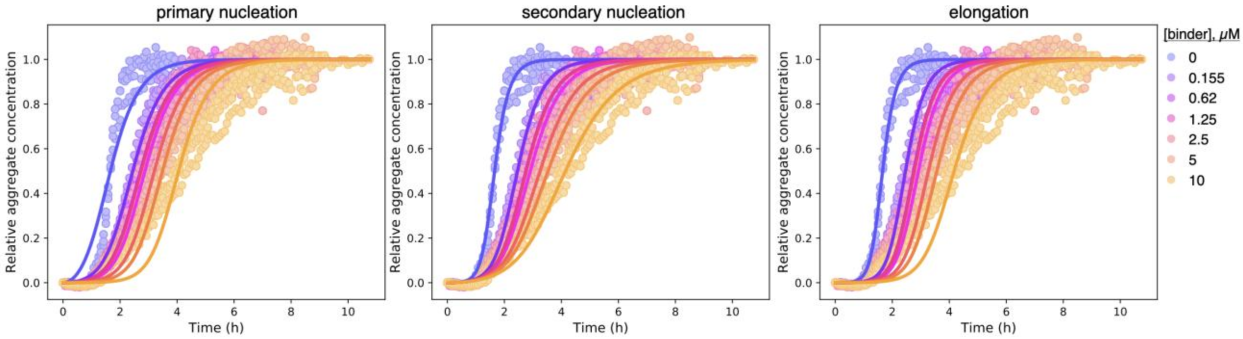

Supplementary Figure 10. ThT fluorescence assays of Aβ42 aggregation in presence of varying concentrations of SXkmer-CRLISFF fit to a selective reduction of primary nucleation, secondary nucleation, or elongation rate constants using Amylofit.[47]

Supplementary Table 1. Key Plasmids used in this work.

Plasmid name	Resistance	Origin	ORF		
			Promoter	*RBS*	*Gene*
pBL071	Spec	ColE1	P_{tet}	sd8[50]	*cCadC-Aβ42*
pBL071b	Spec	ColE1	P_{tet}	sd8	*cCadC-Aβ42 F20S/L35P*
pTW390b	Spec	ColE1	P_{tet}	sd8	*cCadC-Aβ42 ΔE22*
pBL188	Kan	P15a	P_{tac}	SD8	*SXkmer-CRLISFF*
pBL188c	Kan	P15a	P_{tac}	SD8	*SXkmer-CKVWQLL*
pBL188d	Kan	P15a	P_{tac}	SD8	*SXkmer-CFVQLFF*
LBL141z	Kan	P15a	P_{tac}	SD8	*Cfa SICLOPPS* CX_6 *library*
pEP003a1	Kan	P15a	P_{tac}	SD8	*Cfa-(P23L)-CFVQLFF*
pEP003c1	Kan	P15a	P_{tac}	SD8	*Cfa-(P23L)-CRLISFF*
pEP003a	Kan	P15a	P_{tac}	SD8	*Cfa-(P23)-CFVQLFF*
pEP003c	Kan	P15a	P_{tac}	SD8	*Cfa-(P23)-CRLISFF*
pBL215b	Kan	P15a	P_{tac}	SD8	*Cfa-(PL23, H24L/F26L)-CRLISFF*
pBL215c	Kan	P15a	P_{tac}	SD8	*Cfa-(P23L, H72A/T75A)-CRLISFF*
pBL215d	Kan	P15a	P_{tac}	SD8	*Cfa-(P23, H24L/F26L)-CFVQLFF*
pBL215e	Kan	P15a	P_{tac}	SD8	*Cfa-(P23, H72A/T75A)-CFVQLFF*
pEP025	Kan	P15a	P_{tac}	SD8	*Cfa-(P23L)-CRLISFF-6xHis*
pEP028	Kan	P15a	P_{tac}	SD8	*Cfa-CFVQLFF-6xHis*
pEP029	Kan	P15a	P_{tac}	SD8	*Cfa-(P23L)-CFVQLFF-6xHis*
pEP035	Kan	P15a	P_{tac}	SD8	*Cfa-(P23L)-CRLISFF-6xHis*
pEP037	Kan	pMB1	P_{T7}	RBS	*Cfa-(P23L)-ARLISFF-6xHis*
pEP012	Kan	P15a	P_{tac}	SD8	*Cfa-(P23L,* Cfa_C *N36A)-CRLISFF*
pEP013	Kan	P15a	P_{tac}	SD8	*Cfa-(*Cfa_N *C1A, P23L)-CRLISFF*

pEP003c9	Kan	P15a	P_{tac}	SD8	*Cfa-(P23L)-ARLISFF*
pEP014	Kan	P15a	P_{tac}	SD8	*Cfa-(P23, Cfa_C N36A)-CFVQLFF*
pEP015	Kan	P15a	P_{tac}	SD8	*Cfa-(Cfa_N C1A, P23)-CFVQLFF*
pEP003a8	Kan	P15a	P_{tac}	SD8	*Cfa)-AFVQLFF*
pBF001a	Kan	pMB1	P_{T7}	RBS	*6xHis-SXkmer-CRLISFF*
pTW160c[51]	Amp	pSC101	P_{cadBA}	cadB	*luxAB*
pBL140[43]	Amp	pSC101	P_{cadBA}	cadB	*cat*

Supplementary Table 2. Key primers used in this work

Primer name	**Primer Sequence**
BL0671	agttagaugccaccagccc
BL0674	atctaacugcnnknnknnknnknnknnktgcctgtcttacgacacag
